# Treatment delay in status epilepticus – more effective prehospital symptom recognition warranted

**DOI:** 10.1186/s13049-019-0605-7

**Published:** 2019-03-07

**Authors:** Joni J. Sairanen, Anne-Mari Kantanen, Harri T. Hyppölä, Reetta K. Kälviäinen

**Affiliations:** 1 Epilepsy Center, Neuro Center, Kuopio University Hospital, Member of ERN EpiCARE, Kuopio, Finland; 2 Emergency Department, Kuopio University Hospital, Kuopio, Finland; 3 Institute of Clinical Medicine, University of Eastern Finland, Kuopio, Finland

**Keywords:** Status epilepticus, Seizure, Treatment, Delay, Emergency, Paramedic

## Abstract

**Background:**

The outcome of status epilepticus (SE) can be improved by facilitating early recognition and treatment with antiepileptic drugs. The purpose of this study was to analyze the treatment delay of SE in a prospectively recruited patient cohort. Improvements to the treatment process are suggested.

**Methods:**

Consecutive adult patients with SE were recruited in the emergency department of Kuopio University Hospital (KUH) between March 23 and December 31, 2015. SE was defined as a prolonged (> 5 min) epileptic seizure or recurrent tonic-clonic seizures (≥ 3 seizures within any 24 h). Diagnostic and treatment delays and the features of SE were subject to statistical analysis.

**Results:**

We recorded 151 cases of SE during the study period. First-line treatment was initiated outside of hospital in 79 cases (52.3%), with a significantly shorter median delay compared to intrahospital initiation (28 min vs. 2 h 5 min, *p* < 0.001). Forty-six episodes of SE (30.5%) were not recognized during the prehospital phase. The median delay in recognition of tonic-clonic SE (23 min) was significantly shorter than in focal aware (2 h 0 min, *p* = 0.045) or focal impaired awareness SE (2 h 25 min, *p* < 0.001). Second-line treatment was used in 91 cases (60.3%), with a median delay of 2 h 42 min. Anesthesia was used in seven cases (4.6%) with refractory SE, with a median delay of 6 h 40 min.

**Conclusions:**

SE is often not recognized during the prehospital phase of treatment, which delays the initiation of first-line treatment. Intrahospital delay could be reduced by streamlining patient transition between the three lines of treatment.

## Background

Status epilepticus (SE) is an abnormally prolonged epileptic seizure that may cause long-term neurologic complications [1]. The International League Against Epilepsy (ILAE) has defined two critical time points during seizure; seizures that continue beyond time point t_1_ are prolonged and seldom cease spontaneously. After time point t_2_, neuronal death or injury may occur [1]. The case fatality of SE is 7.6–22%, as reported in several studies [2].

The pathophysiology of SE involves the failure of endogenous seizure inhibition or the initiation of a mechanism that leads to an abnormally prolonged seizure [1]. The seizure triggers molecular mechanisms that promote receptor trafficking and altered neuropeptide expression, which cause sustained hyperexcitation in the affected neuronal network [3]. As the seizure duration grows, GABAergic anticonvulsants lose their potency [4] and the risk of refractory seizure [5] and adverse outcome increases [6].

Emergency treatment of SE should be started at time point t_1_, which is at 5 min in tonic-clonic seizures, 10 min in focal impaired awareness seizures, and 10–15 min in absence seizures [1]. The recommended first-line treatment is benzodiazepine administration [7]. Other antiepileptic drugs are frequently needed as second-line treatment. In refractory SE, the patient is usually treated in the intensive care unit (ICU) with general anesthesia as third-line treatment [8]. The one-year mortality in ICU-treated refractory SE is 23%, and an additional 29% show neurologic deficits [9].

Seizure duration is the only modifiable prognostic factor in SE [10]. The patient outcome can be improved by facilitating early and effective treatment with antiepileptic drugs [10]. Early treatment shortens hospital stay and reduces the risk of being admitted to the ICU with refractory SE [11].

Hill et al. recently reviewed 17 observational studies on treatment delay in SE [12]. They noted that 17–64% of patients have a longer than 30-min delay to first-line treatment and only 31–54% receive drug treatment before arrival at the hospital. Most studies on treatment delay of SE are performed retrospectively, which makes it difficult to accurately assess seizure duration and to analyze root causes of delay [12].

### Study objective

The objective of this study was to measure and analyze treatment delay of SE in patients admitted to the emergency department of a major academic Finnish hospital. The study design was prospective to ensure high data quality. Causes of delayed treatment were analyzed and improvements to the treatment process were suggested.

## Methods

### Study design and setting

Our prospectively recruited study cohort consists of consecutive adult patients admitted to the emergency department (ED) of Kuopio University Hospital (KUH) due to prolonged or recurrent epileptic seizures, between March 23 and December 31, 2015. The study was observational and did not modify the management of patients. An investigator (JS) actively searched the patient lists of the ED for eligible study patients. Study data were obtained from hospital records and paper forms used by the emergency medical services (EMS). The use of medical records was authorized by the hospital district in accordance with Finnish legislation. The Committee on Research Ethics of North Savo Hospital District approved the study design.

The study hospital provides the only 24/7 emergency neurology service for a population of 248,000 in the North Savo region of Eastern Finland. The North Savo region has an area of 20,367 km^2^, and travel time to the hospital from the most distant areas served can be up to two hours by car. There are two small regional hospitals in the region and a network of publicly run community health centers. The regional hospitals and health centers provide a 24/7 availability of general practitioners (GPs) but limited diagnostic and treatment options in neurologic emergencies. SE patients are generally referred to KUH, unless do-not-hospitalize (DNH) orders or other special circumstances exist.

The EMS are arranged by KUH for the whole North Savo region with 24 ambulances and a physician-staffed emergency medical helicopter. The ambulance units consist of seven basic (BLS) and seventeen advanced life support (ALS) units. SE is classified as a high-priority mission and the closest unit is dispatched. According to the regional treatment guideline of SE, any ambulance unit can start the treatment of SE with buccal midazolam. After venous access has been obtained, an ALS unit may use intravenous diazepam. Benzodiazepine treatment can be started based on clinical judgement of the paramedics before consulting a physician. In the case of benzodiazepine-resistant seizure with long distance to hospital, a physician-staffed unit will be dispatched with the capability to administer second-line drugs.

### Participants

Study participants were adults (≥ 16 years of age) who fulfilled the operational definition of SE presented in the Finnish Current Care Guideline [13]: a prolonged (> 5 min) epileptic seizure, a seizure cluster (≥ 2 discrete seizures) with no complete interictal recovery, or a recurrent tonic-clonic seizure with three or more isolated seizures within any 24-h period. Patients with postanoxic seizures were excluded. The seizures were recognized clinically, and the diagnosis was always verified by a physician. Electroencephalogram (EEG) confirmation was required for nonconvulsive seizures. Cases of SE were registered independently; we did not exclude cases in which the same study patient was readmitted for SE and again fulfilled the inclusion criteria.

### Measurements

An investigator (JS) collected the data from medical records and structured forms filled by EMS personnel on a preformatted form. The data were stored as a SPSS 24 (IBM Corp, New York) dataset. Treatment delay was measured as time medians from the onset of SE to specific points of the treatment process, except that if the onset of SE was not witnessed, the delays were measured from the moment the patient was discovered. In a recurrent tonic-clonic seizure, the delays were measured from the onset of third seizure, which by operational definition marks the beginning of SE. The time of SE recognition was the moment when a person capable of treating SE (a paramedic, a physician or a caretaker) correctly recognized SE or when seizure activity was found on EEG.

### Analysis

SPSS 24 was used in data analysis. Descriptive statistics were used to report the basic features of the study cohort. The cases were grouped according to seizure type and prehospital or intrahospital initiation of drug treatment. In subgroup analyses, the Mann-Whitney test and the Kruskal-Wallis test were used to find statistically significant differences in treatment delay between the patient groups. Dunn’s test was used in post-hoc analyses and the Bonferroni correction was used to adjust *p*-values. Two-tailed tests were used, and the level of statistical significance was set at *p* < 0.05. In our previous studies, the ratio of tonic-clonic SE to other types of SE has been 60:40, so we estimated that a sample size of 150 would be sufficient to compare the clinically meaningful treatment delays in main seizure types.

## Results

### Characteristics of study cohort

A total of 151 cases of SE in 137 individual patients (mean age: 59.5 years) were recorded. Half of the 137 patients (49.6%) had a prior diagnosis of epilepsy. Cerebrovascular disease (29.9%) and dementia of any etiology (22.6%) were common comorbidities, as well as psychiatric illness with ongoing drug treatment or psychotherapy (34.3%). There was a clinically relevant history of alcohol abuse in one of every three (33.6%) patients. The majority (80.3%) of patients lived independently at home.

Clinical features of the 151 SE cases are summarized in Table 1. Tonic-clonic seizures were the most common (69.5%), followed by focal impaired awareness seizures (14.6%) and focal aware seizures (9.9%). Myoclonic and absence seizures were rare. The most common etiology of a seizure was unknown (25.8%), either with or without diagnosis of pre-existing epilepsy. The second most common etiology was alcohol withdrawal (17.2%) followed by past cerebrovascular accident (15.9%). The usual scene of onset was at home, either with someone else (41.7%) or alone (7.9%). A large proportion of seizures began in a healthcare unit (20.5%) or a nursing home (13.2%). The onset of seizure was not witnessed in 27 cases (17.9%). Forty-seven (31.1%) of the SE episodes had an intermittent course either de novo or because of partial treatment response.

**Table 1:** Clinical features of the 151 SE cases

	N	%
Scene of SE onset		
Home, with someone else	63	41.7
Healthcare unit	31	20.5
Public place	23	15.2
Nursing home	20	13.2
Home, alone	12	7.9
Prison	2	1.3
Seizure etiology		
Acute symptomatic	57	37.7
Alcohol withdrawal	26	17.2
Drug withdrawal	18	11.9
Cerebrovascular accident	6	4.0
CNS infection	3	2.0
Drug toxicity	2	1.3
Metabolic insult	1	0.7
Head trauma	1	0.7
Unknown	39	25.8
Epilepsy with unknown or genetic etiology	24	15.9
Unknown	15	9.9
Remote symptomatic	38	25.2
Previous cerebrovascular accident	24	15.9
Previous brain injury	6	4.0
CNS anomaly	4	2.6
Previous brain surgery	2	1.3
Previous CNS infection	2	1.3
Progressive symptomatic	17	11.3
Degenerative brain disease	9	6.0
Brain tumor	8	5.3
Seizure type before treatment		
Tonic-clonic	105	69.5
Focal, impaired awareness	22	14.6
Focal, aware	15	9.9
Nonconvulsive comatose	5	3.3
Absence	2	1.3
Myoclonic	2	1.3
SE recognition		
In prehospital setting	90	59.6
Paramedic	68	45.0
GP	11	7.3
Caretaker	6	4.0
EMS physician	5	3.3
In hospital	61	40.4
ED neurologist	56	37.1
ED neurologist/clinical neurophysiologist (EEG diagnosis)	5	3.3
Means of transportation after onset of SE		
Ambulance	133	88.1
Already admitted to ED	10	6.6
Private car	5	3.3
Helicopter	3	2.0
Health care unit where first treated		
Kuopio University Hospital	124	82.1
Community health center (no neurologist on call)	21	13.9
Regional hospital (no neurologist on call)	6	4.0

*SE* Status epilepticus, *CNS* Central nervous system, *GP* General practitioner, *EMS* Emergency medical services, *ED* Emergency department, *EEG* Electroencephalogram. Transport by helicopter is arranged by the Helicopter Emergency Medical Service that has a landing pad at Kuopio University Hospital

The onset of SE was outside of KUH in 141 cases (93.4%). A caretaker, paramedic, GP or an EMS physician recognized SE in 90 cases during the prehospital treatment. In forty-six cases, SE was initially not recognized, and in five cases a private car was used to get to hospital. Ten episodes of SE (6.6%) began in the ED. The diagnosis of SE was made by an ED neurologist in 56 cases. In five cases, the diagnosis was made by either an ED neurologist or a clinical neurophysiologist after an acute EEG study was conducted.

### Main results

**Fig. 1:**
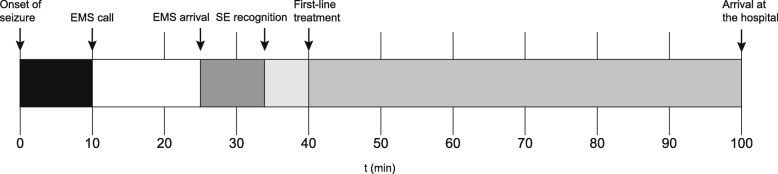
Delay components in the prehospital treatment of status epilepticus. EMS, emergency medical services; SE, status epilepticus. Delay components are shown where their median value (min) falls on the timeline

The measured delay components in the treatment of SE are shown in Table 2. The median delay to EMS call was 10 min, followed by EMS arrival at 25 min. In 14 cases (9.3%), an EMS physician met the patient outside of hospital with a median delay of 48 min. The time of initial SE recognition could be verified in 148 cases (98.0%), and the median SE recognition delay was 34 min. Fig. 1 shows the reported delays on a timeline.

**Table 2:** Treatment delays of SE

Delay component	Median	Range	N	%
EMS call	10 min	0–12 h 4 min	117	77.5
EMS arrival	25 min	0–12 h 31 min	116	76.8
EMS physician arrival	48 min	5 min – 1 h 33 min	14	9.3
SE recognition	34 min	0–67 h 29 min	148	98.0
First-line treatment	40 min	0–48 h 44 min	121	80.1
Arrival at the hospital	1 h 40 min	0–51 h 23 min	150	99.3
Second-line treatment	2 h 42 min	10 min – 71 h 30 min	91	60.3
EEG initiation	5 h 11 min	1 h 41 min – 67 h 29 min	44	29.1
Onset of anesthesia	6 h 40 min	3 h 48 min – 7 h 30 min	7	4.6
BS pattern on EEG	8 h 20 min	5 h 35 min – 9 h 20 min	7	4.6

*SE* Status epilepticus, *EMS* Emergency medical services, *ED* Emergency department, *EEG* electroencephalogram, *BS* burst suppression

Time parameters are counted from the onset of SE. In cases where the onset of SE was not witnessed, the parameters are counted from when the patient was discovered

First-line treatment was given in 121 cases (80.1%), with a median delay of 40 min. Reasons why first-line treatment was not given include (1) spontaneous recovery in 17 cases, (2) the decision to proceed straight to second-line treatment in 11 cases and (3) consistency issues with the treatment guideline in two cases: one patient was given intravenous midazolam, and one patient received oral diazepam mixture to treat alcohol withdrawal symptoms and prevent the recurrence of a tonic-clonic seizure.

Of all 121 first-line treatments given, 79 (65.3%) were initiated outside of hospital. Prehospital initiation of drug treatment was associated with significantly shorter first-line treatment delay compared to intrahospital initiation (28 min vs. 2 h 5 min, *p* < 0.001).

The median delay to arrival at the hospital was 1 h 40 min. Second-line treatment was given in 91 cases (60.3%), with a median delay of 2 h 42 min. An acute EEG study was conducted on 44 subjects (29.1%) at the median time point of 5 h 11 min. Seven patients (4.6%) received third-line treatment for refractory SE, and the median delay to anesthesia was 6 h 40 min.

### Treatment delay in different types of seizures

**Fig. 2:**
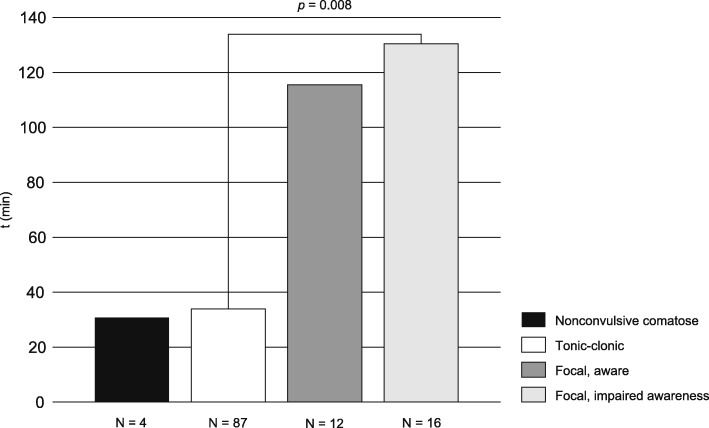
Comparison of median first-line treatment delay in the four most common seizure types. In pairwise analysis (Dunn-Bonferroni test), the treatment delay was significantly shorter in tonic-clonic seizures compared to focal impaired awareness seizures (32 min vs. 2 h 10 min, *p* = 0.008)

Across groups with different seizure types, a Kruskal-Wallis test revealed statistically significant differences in the following delay components: time to EMS call (*p* = 0.002), time to SE recognition (*p* < 0.001), time to first-line treatment (*p* < 0.001) and time to second-line treatment (*p* = 0.015). In pairwise analysis (Dunn-Bonferroni test), the delay to SE recognition was significantly shorter in a tonic-clonic seizure (23 min) than in a focal aware (2 h 0 min, *p* = 0.045) or a focal impaired awareness seizure (2 h 25 min, *p* < 0.001). Figure 2 illustrates how the first-line treatment time in a tonic-clonic seizure (32 min) was significantly shorter than in a focal impaired awareness seizure (2 h 10 min, *p* = 0.008). The time to EMS call was significantly shorter in a comatose patient with a nonconvulsive seizure (2 min) than in a patient with a focal impaired awareness seizure (28 min, *p* = 0.042). Other pairwise analyses yielded statistically insignificant results.

## Discussion

In this study, treatment delays of SE were investigated. Long delays were found in both the prehospital and intrahospital phases of the treatment process. Our measurements of first-line, second-line and third-line treatment delay are in line with those summarized earlier in the review by Hill et al. [12] except that the delay to third-line treatment, 6 h 40 min, is noticeably longer in our study. There is considerable room for improvement in earlier recognition and treatment of SE, especially in nonconvulsive seizures.

Timely administration of first-line drugs can stop the seizure before it becomes refractory to treatment [3] and before neurologic damage occurs [7]. The t_2_ points of tonic-clonic SE and focal SE with impaired awareness are 30 min and > 60 min, respectively [1]. Such a short time window usually requires initiation of drug treatment outside of hospital, either by EMS personnel or, in some cases with refractory epilepsy, the patient’s caretaker. Home prescriptions of rescue medication are of limited help because (1) only half of the patients with SE have a prior diagnosis of epilepsy, (2) not all patients with epilepsy have a predictable need for rescue medication and (3) not all patients with refractory epilepsy can be prescribed rescue medication.

The prehospital phase of treatment in our cohort was time-consuming: its median duration was 1 h 40 min. Two earlier Finnish studies reported a median prehospital delay of 1 h 45 min [14] and 2 h 4 min [15]. The median EMS call delay (10 min) was short, so most of the overall delay was due to movement of the EMS unit, treatment given on-site and patient transport. Over half of our cases (52%) received first-line treatment outside of hospital with the median delay of 28 min, which is within the t_2_ of tonic-clonic SE. In contrast, if the first-line treatment was initiated only after arrival at hospital, its median delay was 2 h 5 min. This highlights the role of EMS personnel and caretakers in successful symptom recognition and rapid treatment initiation.

Difficulty recognizing SE may delay treatment initiation to the point when benzodiazepine medication is no longer effective. Across all 151 cases, the median delay to SE recognition was 34 min, and 46 cases of SE were initially missed during the prehospital phase. We noticed a tendency towards longer delay in focal seizures. In focal aware and focal impaired awareness SE, the recognition delays were 2 h 0 min and 2 h 25 min, respectively, and the seizure symptoms were often missed before hospital arrival. Some delay could possibly have been eliminated if the patient’s history of unusual seizures had been known to the EMS responders. EMS personnel and GPs should also suspect nonconvulsive SE more readily when encountering obtunded elderly patients. In uncertain prehospital situations the correct place of follow-up care is a hospital with available acute EEG.

Second-line treatment was given in 91 cases (60.3%), and the associated delay of 2 h 42 min is alarming. Seven patients (4.6%) received third-line treatment for refractory SE, and the median time to anesthesia was 6 h 40 min. These findings suggest problems in the transition between the three lines of treatment. Variability in the dosing of benzodiazepines, such as too many treatment attempts before moving on to the next line of treatment, has been noted as a potential source of delay [12]. Sometimes the resolution of seizure remains uncertain after the administration of second-line drugs. If the patient’s ongoing seizure is not correctly recognized, the acute EEG is ordered too late and the third-line treatment of refractory SE is needlessly delayed.

The phrase “time is brain” is well-known in stroke research. The diagnostic and treatment delays of acute stroke can be greatly reduced by developing EMS-ED cooperation and streamlining intrahospital care [16]. Some measures that have proved themselves valuable in stroke should be used in the treatment of SE as well. They include an EMS pre-notice before hospital arrival and a rapid neurologic evaluation in a prearranged place, for example the resuscitation room, with a preordered laboratory test package. Ictal symptoms and the given antiepileptic drugs with appropriate time tags should be documented systematically so that the need for second- or third-line medication can be noticed earlier.

The limitations of this study include the relatively small cohort size and possible bias in recording the exact time points of seizure onset and treatment procedures. Some of our subgroup analyses lack power because there were only few cases with a rare seizure type. The data were collected during a nine-month period in a single Finnish tertiary hospital. Our cohort does not include those SE patients in our hospital district who were not treated in KUH emergency department due to special circumstances, for example do-not-hospitalize (DNH) orders. The local EMS guidelines and especially geography have influenced the measured treatment delays in this study and our results may not be readily generalized to other populations with different geographical distribution and access to care even in Finland.

## Conclusion

In summary, significant delay occurs in all phases of treatment in SE. Early recognition of SE is crucial to ensure rapid treatment initiation, preferably during the prehospital phase of the treatment process. EMS personnel must be prepared to treat all types of epileptic seizures promptly with the recommended first-line drugs. EMS personnel and GPs must be educated about the possibility and clinical signs of a focal seizure. Rescue medication should be made available to any eligible patient with a caretaker and a history of uncontrolled seizures.

## Abbreviations

ALS: Advanced life support
BLS: Basic life support
CNS: Central nervous system
DNH: do-not-hospitalize
ED: Emergency department
EEG: electroencephalogram
EMS: Emergency medical services
GP: General practitioner
ICU: Intensive care unit
ILAE: International League Against Epilepsy
KUH: Kuopio University Hospital
SE: Status epilepticus
